# A New Restriction Endonuclease-Based Method for Highly-Specific Detection of DNA Targets from Methicillin-Resistant *Staphylococcus aureus*

**DOI:** 10.1371/journal.pone.0097826

**Published:** 2014-05-15

**Authors:** Maria W. Smith, Andrei L. Ghindilis, Ihab A. Seoudi, Kenneth Smith, Rosalind Billharz, Holly M. Simon

**Affiliations:** 1 Center for Coastal Margin Observation & Prediction, and Institute of Environmental Health, Oregon Health & Science University, Portland, Oregon, United States of America; 2 Cascade Biosystems, Inc., Colfax, Wisconsin, United States of America; 3 Hamad Medical Corporation, Doha, Qatar; 4 Pacific Lutheran University, Department of Biology, Tacoma, Washington, United States of America; Kliniken der Stadt Köln gGmbH, Germany

## Abstract

PCR multiplexing has proven to be challenging, and thus has provided limited means for pathogen genotyping. We developed a new approach for analysis of PCR amplicons based on restriction endonuclease digestion. The first stage of the restriction enzyme assay is hybridization of a target DNA to immobilized complementary oligonucleotide probes that carry a molecular marker, horseradish peroxidase (HRP). At the second stage, a target-specific restriction enzyme is added, cleaving the target-probe duplex at the corresponding restriction site and releasing the HRP marker into solution, where it is quantified colorimetrically. The assay was tested for detection of the methicillin-resistant *Staphylococcus aureus* (MRSA) pathogen, using the *mecA* gene as a target. Calibration curves indicated that the limit of detection for both target oligonucleotide and PCR amplicon was approximately 1 nM. Sequences of target oligonucleotides were altered to demonstrate that (i) any mutation of the restriction site reduced the signal to zero; (ii) double and triple point mutations of sequences flanking the restriction site reduced restriction to 50–80% of the positive control; and (iii) a minimum of a 16-bp target-probe dsDNA hybrid was required for significant cleavage. Further experiments showed that the assay could detect the *mecA* amplicon from an unpurified PCR mixture with detection limits similar to those with standard fluorescence-based qPCR. Furthermore, addition of a large excess of heterologous genomic DNA did not affect amplicon detection. Specificity of the assay is very high because it involves two biorecognition steps. The proposed assay is low-cost and can be completed in less than 1 hour. Thus, we have demonstrated an efficient new approach for pathogen detection and amplicon genotyping in conjunction with various end-point and qPCR applications. The restriction enzyme assay may also be used for parallel analysis of multiple different amplicons from the same unpurified mixture in broad-range PCR applications.

## Introduction

PCR-based nucleic acid detection techniques have become the standard methodology in clinical and research microbiology and molecular diagnostics of infectious diseases (for reviews see [1], [2], [3]). PCR-amplified DNA targets of interest (amplicons) may be quantified either simultaneously with DNA synthesis as in quantitative real-time PCR (qPCR), or after completion as in end-point applications. A large number of methods are used for amplicon detection, most involving fluorogenic-based systems and complex instrumentation. Other approaches have been developed, including electrochemical detection of amplicons in a microarray-based format [4], surface plasmon resonance, sandwich hybridization assays (SHAs) or the fluorescence *in situ* hybridization (FISH) test [2], [5]. Direct DNA sequencing can also be used for amplicon characterization. However, it is still a relatively expensive and time-consuming approach. Thus, qPCR applications are significantly more expensive than many conventional techniques, such as culturing and immunoassays [2]. Moreover, many conventional methods provide for simultaneous detection of multiple organisms of interest together with pathogen characterization and genotyping [6]. In contrast, PCR has proven to be challenging to multiplex because of both primer and probe design concerns. As a result, several modules for amplicon characterization are required, further increasing costs. Amplicon genotyping is especially important for pathogen detection in complex environmental microbial communities. An attractive approach involves the use of ‘universal’ primers (designed from conserved sequences) to generate a mixed population of amplicons (‘broad-ranged PCR’) [7]. This approach, however, requires the development of additional, low-cost and rapid techniques to analyze the resulting mixture of PCR products simultaneously [2].

We developed a novel amplicon genotyping technique, and have tested the approach for detection of an important pathogen: methicillin-resistant *Staphylococcus aureus* (MRSA). *S. aureus* is the most common cause of hospital-acquired infections with an estimated annual impact between 12,000 and 18,650 patient deaths per year, 2.7 million extended hospitals days and $9.5 billion excess costs, in the United States alone [8], [9]. This pathogen developed resistance to penicillin and newer β-lactam antimicrobial drugs (*e.g.* methicillin), and as a result MRSA epidemics have spread widely in hospitals and throughout ordinary community settings (for a review see [10]). The pathogen was also recently isolated from marine water and intertidal beach sand from US West Coast public marine beaches [11]. Similarity between the environmental strains and hospital pathogens suggested that public beaches may serve as reservoirs for transmission of MRSA to beach visitors, and for exchange of antibiotic resistance genes among staphylococci and related genera [11].

Current MRSA screening is based on a combination of culturing, qPCR-based assays and coagulase tests to determine genotype and strain characteristics of the pathogen [11], [12], [13], [14], [15]. MRSA-specific gene targets are located on a mobile genetic element, the Staphylococcal cassette chromosome (SCC) (reviewed in [16]). The main target for qPCR assays, the *mecA* gene, is responsible for the antibiotic resistance phenotype, and encodes a peptidoglycan transpeptidase which functions in cell wall biosynthesis when the three other essential transpeptidases have been inactivated by β-lactam antibiotics [11], [12], [13], [14], [15], [16], [17], [18]. Recent studies showed high sensitivity of qPCR-based methods used for *mecA* detection, *i.e.* the IDI-MRSA kit (GeneOhm Sciences Canada, Ste-Foy, QC, Canada) and GenoType MRSA Direct (Hain Lifescience, Nehren, Germany) [19], [20]. In addition to classical qPCR, novel methods are being developed, including a droplet digital PCR (ddPCR), which is a next-generation emulsion-based endpoint PCR assay [21]. The main focus of using PCR for MRSA screening is to reduce the assay turnaround time to 2–4 h (from 24–48 h required for culturing techniques), which in turn could drastically decrease the incidence of MRSA disease due to fast decision making in hospital settings [22]. Several factors complicate the PCR applications, including high genetic variability and continuous emergence of new MRSA strains, and the presence of cross-reactive sequences in methicillin-susceptible staphylococci, such as *S. epidermidis*. Thus, the ability to perform extensive pathogen genotyping of multiple loci is crucial. Otherwise, the standard culturing methods are still considered necessary for confirmation of qPCR results [20].

**Figure 1:**
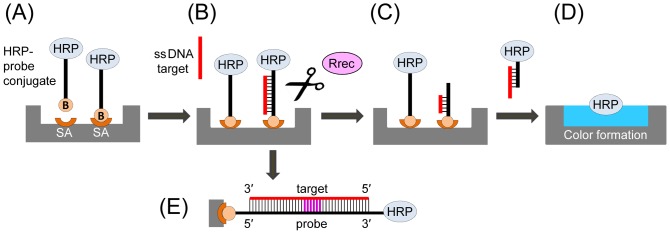
General schematic of the restriction enzyme assay. (**A**) Surface immobilization of HRP conjugated to an oligonucleotide probe specific for a target gene of interest. (**B**) The target DNA (an oligonucleotide or a denatured PCR amplicon) is hybridized to the immobilized probe. (**C**) Addition of a restriction enzyme (Rrec) that recognizes and cleaves the target-probe ds DNA hybrid, resulting in release of the HRP marker into the reaction solution. (**D**) The reaction solution is transferred into a new well and mixed with an HRP substrate for colorimetric detection. For each target DNA molecule one HRP molecule is released, resulting in a linear dependence of the signal on the target DNA concentration. (**E**) Detailed schematic of the double stranded target-probe DNA duplex, with the specific restriction site shown in purple. HRP, horseradish peroxidase; B, biotin; SA, streptavidin.

We describe a new approach for amplicon detection and genotyping based on specific enzymatic digestion of a target DNA. Enzymatic digestion with RNase H was previously proposed for detection of the *mecA* gene using a colorimetric enzyme immunoassay referred to as “cycling probe technology” [23]. Our approach is based on the selective cleavage reaction performed by restriction endonucleases, namely Class II restrictases. These enzymes have nearly absolute sequence specificity for a particular double-stranded (ds) DNA sequence (typically, a palindromic site composed of two equivalent half-sites with the total length of 4–8 bp) [24], [25]. Restriction enzymes use linear diffusion or “sliding” to move along a DNA duplex, with cleavage occurring only when the protein–DNA interface is correctly assembled at the proper recognition site [24]. Previously, these enzymes have been used for characterization of PCR amplicons, generating a unique pattern of DNA fragments to serve as a fingerprint when gel electrophoresis is performed (see [1]). Our assay does not require gel electrophoresis, and it has only two main steps: the first is hybridization of the target DNA (*i.e.* an amplicon) to immobilized complementary oligonucleotide probes (Fig. 1B). The immobilized probes carry a molecular marker, horseradish peroxidase (HRP), attached to the end of the oligonucleotide that is free in solution (Fig. 1A, E). After completion of the target-probe hybridization, the second step involves the addition of a target-specific restriction enzyme, which cleaves the DNA duplex and releases the HRP marker into solution (Fig. 1C). The release can only occur if the target binds to the probe, which results in creation of the cognate site for recognition and restriction by the enzyme [25]. With cleavage, one HRP molecule is released per each target molecule. Upon assay completion (5 to 60 min), the reaction mixture is transferred to a detection chamber for colorimetric quantitation of the released HRP (Fig. 1D). Because this assay involves two biorecognition steps, which are (i) target-probe DNA duplex formation; and (ii) subsequent sequence-specific cleavage of the duplex by a restriction enzyme, it is advantageous compared to standard amplicon detection techniques with respect to increased specificity and significantly reduced probability of false positives. Furthermore, it does not require expensive fluorescent reagents, instead relying on a variety of standard HRP substrates that can be used in a multitude of low cost detection formats. In its current application, this technique can be coupled with end-point PCR, and/or used in a near real-time format for amplicon detection and quantitation.

## Materials and Methods

### Oligonucleotide probes and targets

Table 1 provides a full list of oligonucleotides used in this work, all purchased from Eurofins MWG/Operon (Huntsville, AL). The hybridization probe, 40-mer oligonucleotide MCA-BG, 5′-Biotin-CAATTAAGTTTGCATAAGATCTATAAATATCTTCTTTATG-Thiol-3′, was designed using the conserved *mecA* gene sequences flanking the *Bgl*II restriction site with the recognition site positioned near the probe center. The probe sequence was checked for the absence of stable secondary structure formation using DINAMelt software [26]. The probe was biotin-modified at the 5′ end for surface attachment, and thiol-modified at the 3′ end for HRP conjugation (described below).

**Table 1:** The oligonucleotide probe and targets used in the current study.

Construct	Designation	Sequence	Length	Match^1^	Tm(°C)^2^
Probe	MCA-BG	CAATTAAGTTTGCATA**AGATCT**ATAAATATCTTCTTTATG	40	n/a	n/a
Positive control target	40-mer	CATAAAGAAGATATTTAT**AGATCT**TATGCAAACTTAATTG	40	40	68.1
Restriction site mutations	rs19G	CATAAAGAAGATATTTAT**gGATCT**TATGCAAACTTAATTG	40	39	67.9
Restriction site mutations	rs19+24	CATAAAGAAGATATTTAT**gGATCc**TATGCAAACTTAATTG	40	38	66.6
Restriction site mutations	rs24G	CATAAAGAAGATATTTAT**AGATCg**TATGCAAACTTAATTG	40	39	66.6
Single mutation	C29G	CATAAAGAAGATATTTAT**AGATCT**TATGgAAACTTAATTG	40	39	69
Single mutation	T27G	CATAAAGAAGATATTTAT**AGATCT**TAgGCAAACTTAATTG	40	39	68.2
Single mutation	T25G	CATAAAGAAGATATTTAT**AGATCT**gATGCAAACTTAATTG	40	39	66.9
Single mutation	T15G	CATAAAGAAGATATgTAT**AGATCT**TATGCAAACTTAATTG	40	39	65.4
Single mutation	T18G	CATAAAGAAGATATTTAg**AGATCT**TATGCAAACTTAATTG	40	39	68.1
Double mutations	GA27	CATAAAGAAGATATTTAT**AGATCT**TAgaCAAACTTAATTG	40	38	67.7
Double mutations	GG25	CATAAAGAAGATATTTAT**AGATCT**ggTGCAAACTTAATTG	40	38	70.2
Double mutations	GG14	CATAAAGAAGATATggAT**AGATCT**TATGCAAACTTAATTG	40	38	64.7
Double mutations	GG18	CATAAAGAAGATATTTgg**AGATCT**TATGCAAACTTAATTG	40	38	67.9
Triple mutations	GGG16	CATAAAGAAGATATTggg**AGATCT**TATGCAAACTTAATTG	40	37	67.9
Triple mutations	GGG15	CATAAAGAAGATATgggT**AGATCT**TATGCAAACTTAATTG	40	37	64.3
Triple mutations	GGG14	CATAAAGAAGATAgggAT**AGATCT**TATGCAAACTTAATTG	40	37	64.8
Triple mutations	GGG13	CATAAAGAAGATgggTAT**AGATCT**TATGCAAACTTAATTG	40	37	64.3
Triple mutations	GGG12	CATAAAGAAGAgggTTAT**AGATCT**TATGCAAACTTAATTG	40	37	64.5
Triple mutations	CGG11	CATAAAGAAGcggTTTAT**AGATCT**TATGCAAACTTAATTG	40	37	64.8
Triple mutations	GGG25	CATAAAGAAGATATTTAT**AGATCT**gggGCAAACTTAATTG	40	37	67.3
Triple mutations	GGA26	CATAAAGAAGATATTTAT**AGATCT**TggaCAAACTTAATTG	40	37	67.4
Triple mutations	ACG27	CATAAAGAAGATATTTAT**AGATCT**TAacgAAACTTAATTG	40	37	67.5
Triple mutations	AAG28	CATAAAGAAGATATTTAT**AGATCT**TATaagAACTTAATTG	40	37	69.2
Triple mutations	AGG29	CATAAAGAAGATATTTAT**AGATCT**TATGaggACTTAATTG	40	37	68.8
Triple mutations	GGG30	CATAAAGAAGATATTTAT**AGATCT**TATGCgggCTTAATTG	40	37	66.8
Target length	30-mer	GAAGATATTTAT**AGATCT**TATGCAAACTTA	30	30	65.8
Target length	22-mer	ATATTTATAGATCTTATGCAAA	22	22	58.6
Target length	20-mer	TATTTAT**AGATCT**TATGCAA	20	20	57.3
Target length	20/40	atgcctactacTATTTAT**AGATCT**TATGCAAgacctccat	40	20	61.3
Target length	18-mer	ATTTATAGATCTTATGCA	18	18	55.4
Target length	16-mer	TTTAT**AGATCT**TATGC	16	16	52.4
Target length	16/40	atgcctactacgtTTTAT**AGATCT**TATGCcggacctccat	40	16	60.5
Target length	14-mer	TTATAGATCTTATG	14	14	49.8
Target length	12-mer	TAT**AGATCT**TAT	12	12	46.2
Target length	12/40	atgcctactacgtacTAT**AGATCT**TATaacggacctccat	40	12	60.6
Target length	6-mer	**AGATCT**	6	6	43.4
Position and loops	5′-C	CATAAAGAAGATATTTAT**AGATCT**TAT	27	27	64
Position and loops	rs3′+0-A	CATAAAGAAGATATTTAT**AGATCT** atgaacggacctccat	40	24	66
Position and loops	rs3′+0-G	CATAAAGAAGATATTTAT**AGATCT** gccaacggacctccat	40	24	66
Position and loops	rs3′+3	CATAAAGAAGATATTTAT**AGATCT**TATaacggacctccat	40	27	66.5
Position and loops	rs3′+5	CATAAAGAAGATATTTAT**AGATCT**TATGCcggacctccat	40	29	66.6
Position and loops	5′-L-5	CATAAAGAAGATATTcgtacTAT**AGATCT**TAT	32	27	62.4
Position and loops	5′-L-10	CATAAAGAAGATATTtactacgtacTAT**AGATCT**TAT	37	27	64.8
Position and loops	3′-C	TAT**AGATCT**TATGCAAACTTAATTG	25	25	63
Position and loops	rs5′+0	atgcctactacgtacctg**AGATCT**TATGCAAACTTAATTG	40	22	63
Position and loops	rs5′+3	atgcctactacgtacTAT**AGATCT**TATGCAAACTTAATTG	40	25	63.5
Position and loops	rs5′+5	atgcctactacgtTTTAT**AGATCT**TATGCAAACTTAATTG	40	27	63.9
Position and loops	3′-L-5	TAT**AGATCT**TATaacggGCAAACTTAATTG	30	25	58.2
Position and loops	3′-L-10	TAT**AGATCT**TATaacggacctcGCAAACTTAATTG	35	25	60.9

Capital letters show sequences that are cognate between a target oligonucleotide and the probe, with the restriction site shown in bold.

1 The total length of a target sequence that is complementary to the 40-mer probe MCA-BG.

2 Tm was calculated for a target-probe hybrid in PBS (150 mM Na^+^).

### Generation of target dsDNA amplicons using PCR

Purified MRSA genomic DNA was purchased from ATCC (http://www.atcc.org/) (Manassas, Virginia, USA, cat. # BAA-1717D-5). The strain TCH1516 (USA300-HOU-MR) originated as a clinical isolate from an adolescent patient with severe sepsis syndrome, and was classified as sequence type 8 (ST8) [27]. The *mecA* amplicon (196 bp) was generated as described in the literature [15], using the primers: MCA-For, 5′-GGCAATATTACCGCACCTCA-3′ (starting at position 1644 of *mecA* gene alignment), and MCA-Rev, 5′-GTCTGCCACTTTCTCCTTGT-3′ (starting at position 1820). The PCR reaction mixture was combined from 25 µL of iQ Supermix (Bio-Rad Laboratories, Inc., Hercules, CA), 1 µL of both forward and reverse primers (20 µM), 0.01 to 10 ng of template strain TCH1516 genomic DNA and nuclease-free water to a total reaction volume of 50 µL. PCR was performed using one cycle of denaturation at 95°C for 5 min, followed by 35 cycles of denaturation at 95°C for 30 sec, annealing at 55°C for 30 sec, and extension at 72°C for 90 sec, with the final extension step at 72°C for 7 min. Aliquots of amplicons were analyzed by agarose gel electrophoresis for the presence of a single band of 196 bp. When necessary, the amplicon was purified using QIAquick PCR Purification Kit (Qiagen, Valencia, CA), and DNA concentrations were measured with a NanoDrop 3300 Fluorospectrometer with the PicoGreen reagent (Thermo Scientific, Wilmington, DE).

### Real-time qPCR

Real-time qPCR was conducted using a MyiQ Real-Time qPCR detection system (Bio-Rad). The reaction mixture was combined from 12.5 µL of iQ SYBR Green Supermix (Bio-Rad), 0.25 µL of both forward and reverse primers (20 µM), 0.01 to 1 ng of template DNA and nuclease-free water to a total reaction volume of 25 µL. The thermocycling was performed as described above. Negative controls were run using nuclease-free water in place of template. Melting curves were visually inspected to check for a single peak at the expected melting temperature using MyiQ software, (v. 1.0.410, BioRad, USA). After PCR completion, the amplicon presence was detected by gel electrophoresis.

### HRP conjugation to oligonucleotide probes

Horseradish peroxidase (HRP) (Thermo Fisher Scientific Inc., Rockford, IL) was activated for conjugation by introducing maleimide groups with the sulfosuccinimidyl-4-(N-maleimidomethyl)cyclohexane-1-carboxylate (Sulfo-SMCC) reagent (Thermo Fisher Scientific), according to a published technique [28] with some modifications. The reaction was carried out in 1X phosphate buffered saline (PBS) (diluted from 10X stock solution (Ambion/Thermo Fisher Scientific)). First, 100 µL of 2 mg/mL HRP solution in PBS was treated with 5 µL of 10 mg/mL (23 mM) Sulfo-SMCC solution in dimethylformamide (DMF). The reaction was incubated at room temperature for 2 h, and then applied twice to Micro Bio-Spin columns with Bio-Gel P-6 (P-6 column) (Bio-Rad, Hercules, CA) to remove the excess of Sulfo-SMCC.

To make sure of the reduced state of thiol groups on the 3′ end of the probe MCA-BG the oligonucleotides were treated using dithiothreitol (DTT) (Thermo Fisher Scientific). In total, 100 µL of 10 µM oligonucleotide solution in nuclease-free water was treated with 4 µL of 500 mM DTT solution in water, and then incubated at room temperature for 3 h. The reaction mixture was purified from the excess of DTT by applying twice to P-6 columns (Bio-Rad).

Finally, we mixed together equal volumes (90 µL each) of the purified SH-modified oligonucleotide solution and activated HRP, and incubated the reaction at 4°C overnight. The resultant HRP-oligonucleotide conjugate, MCA-BG-HRP contained 5 µM oligonucleotide concentration with an excess of unbound HRP. The preparation was used directly for surface immobilization.

### Restriction enzyme assay protocol

For surface immobilization of the HRP-probe through streptavidin-biotin interactions, 30 µL of 50 nM dilution of MCA-BG-HRP conjugate in PBS (1∶100 dilution of 5 µM stock) was applied to each well of a streptavidin-pre-coated 96-microwell plate (Thermo Scientific). The plate was incubated at 4°C overnight, and then washed extensively at room temperature to remove all unbound HRP and conjugate: 6 times with PBS supplemented with 0.05% Tween-20 (PBST), followed by 2 times with PBS.

For target hybridization to the surface-immobilized HRP-probes, working solutions of target oligonucleotides (0–100 nM) were prepared in PBS. The positive control was the fully complementary target 40-mer, and negative control was PBS without oligonucleotides added. Test and control target solutions were added to the wells (20 µL per well) coated with the HRP-probes. Hybridization was performed at 37°C for 30–60 min with gentle shaking (100 rpm), and unbound targets were removed by washing 6X with PBST and 2X with PBS.

Purified amplicons were diluted in PBS as described above for oligonucleotide targets. To use unpurified dsDNA amplicons, the PCR reaction mixture was collected either at the end-point or at an intermediate cycle during PCR. Serial dilutions were made using the pre-cycling PCR mixture (containing the primers and template, but no amplicon). When required, the heterologous mouse genomic DNA (kindly provided by Dr. R. Stephen Lloyd, OHSU) was added as the last step to each dilution, at 100 ng per well.

The test samples containing dsDNA targets (both purified amplicons and whole PCR mixtures) were denatured by heating at 95°C for 5 min followed by incubation on ice for 2 min, and then immediately added to the wells coated with the HRP conjugate for target-probe hybridization. Hybridization was performed at 37°C for 30 min with gentle shaking (100 rpm), and unbound targets were removed by washing as described above for oligonucleotide targets.

The restriction enzyme cleavage of the hybridized target-probe dsDNA was done using 20 µL of the reaction mixture per well. The mixture contained 1∶10 dilution of 10X NEBuffer 3 in nuclease-free water and 0.5 U/µL (1∶20 dilution of the stock) of *Bgl*II restriction enzyme (New England Biolabs, Ipswich, MA). The restriction protocol recommended by the manufacturer (New England Biolabs) was used with the omission of bovine serum albumin (BSA) from the reaction mixture (since BSA presence is known to increase the HRP substrate oxidation background). The restriction reaction was incubated at 37°C for 1 hour with gentle shaking (100 rpm). Finally, to quantify HRP released due to the restriction cleavage, each reaction mixture was transferred to a new ELISA plate well containing 100 µL of the BioFX TMB One Component HRP Microwell Substrate (SurModics, Eden Prairie, MN). The HRP-generated signal was quantified by the blue color formation measured colorimetrically at the wavelength of 655 nM, using an iMark Microplate Reader (Bio-Rad).

### Experimental design and data analysis

OD_655_ measurements were subjected to background subtraction using the corresponding negative control values. For target oligonucleotides and purified amplicons that were hybridized to probes in PBS, the negative controls were prepared from PBS with no targets added. For applications involving non-purified amplicons, the negative control was the unpurified PCR mixture (complete with the primers and template), stored on ice for the duration of the experiment without cycling. Replicates of negative control values (at least 4 replicates per experiment) were used to calculate the mean background values, which were then used for subtraction. Experiments were performed in duplicate or triplicate, with the replicate values used for calculation of mean and standard deviation for each target. For calibration curves, the background-subtracted mean OD_655_ values were plotted against the target concentrations.

The data were additionally normalized to construct calibration curves for the direct comparison of purified versus non-purified amplicons. For each dilution series, we specified the maximum background-corrected signal (generated with the highest 100 nM concentration of target oligonucleotide) as 100%, and expressed all other values in the series as the percentages of the maximum.

A different normalization approach was used for the large-scale comparison of mutant and partially-cognate oligonucleotide targets, since the number of targets (48) was too high to assay in the same experiment. For each oligonucleotide, a series of 4 dilutions (1.6, 6.3, 25, and 100 nM) was prepared. For each series, an integrated signal was calculated as the sum of background-subtracted OD_655_ values obtained in the replicate assays with the 4 dilutions. Next, the mean and standard deviation were calculated using the integrated signals. The same approach was used for the fully cognate 40-mer target positive control, which was assayed in parallel in all experiments. The integrated values for the tested oligonucleotides were used to calculate the HRP signal percentages relative to the positive control (designated as 100%). This approach allowed us to compare across experiments.

## Results

### Hybridization probe design for the MRSA-specific *mecA* gene

The *mecA* gene sequences from members of the *Staphylococcus* genus were collected using the Integrated Microbial Genomes (IMG) web site of DOE Joint Genome Institute (http://img.jgi.doe.gov/). The sequence alignment in Clustal W [29] showed very high conservation (nearly 100% identity over 2 kb length) of this gene among the MRSA strains. Based on the literature, we selected a *mecA* gene fragment commonly used for qPCR [15], and built a detailed restriction map of the predicted amplicon (minus the primers) of 175 bp in length. In total, 6 Class II restriction enzymes (various isoschizomers recognizing the same sequence were considered as one enzyme) had single restriction sites within the amplicon (data not shown). One of them, *Bgl*II, had a relatively long, 6 bp palindromic restriction site AGATCT. The amplicon sequences flanking the *Bgl*II site from both sides had relatively high sequence complexity and did not contain repeat sequences or form stable secondary structures (that may impede the target-probe hybridization). Thus, a 40-mer probe, designated MCA-BG, was designed from the amplicon sequence with the *Bgl*II site in the center (5′-N_16_-AGATCT-N_18_-3′). The probe was modified with biotin at the 5′ end for surface attachment, and a thiol group was added to the 3′ end for conjugation to the molecular marker HRP.

### Restriction enzyme assay design and calibration curve analysis

The general scheme of the proposed restriction enzyme assay is shown in Figure 1. The oligonucleotide probe was conjugated to HRP to generate the MCA-BG-HRP conjugate. The conjugate was attached to the streptavidin coating of ELISA plate wells via the 5′ biotin (Fig. 1A). Next, a single-stranded (ss) target DNA (an oligonucleotide or a denatured PCR amplicon) was hybridized to the immobilized probes (Fig. 1B). Then, a restriction reaction was carried out using the *Bgl*II enzyme, which was specific for the target-probe ds DNA hybrid (Fig. 1C). The restriction enzyme cleaved its cognate site which was formed by the DNA hybrid, releasing the HRP marker into the reaction solution. The reaction solution was transferred into a new well and mixed with an HRP substrate for colorimetric detection (Fig. 1D).

**Figure 2:**
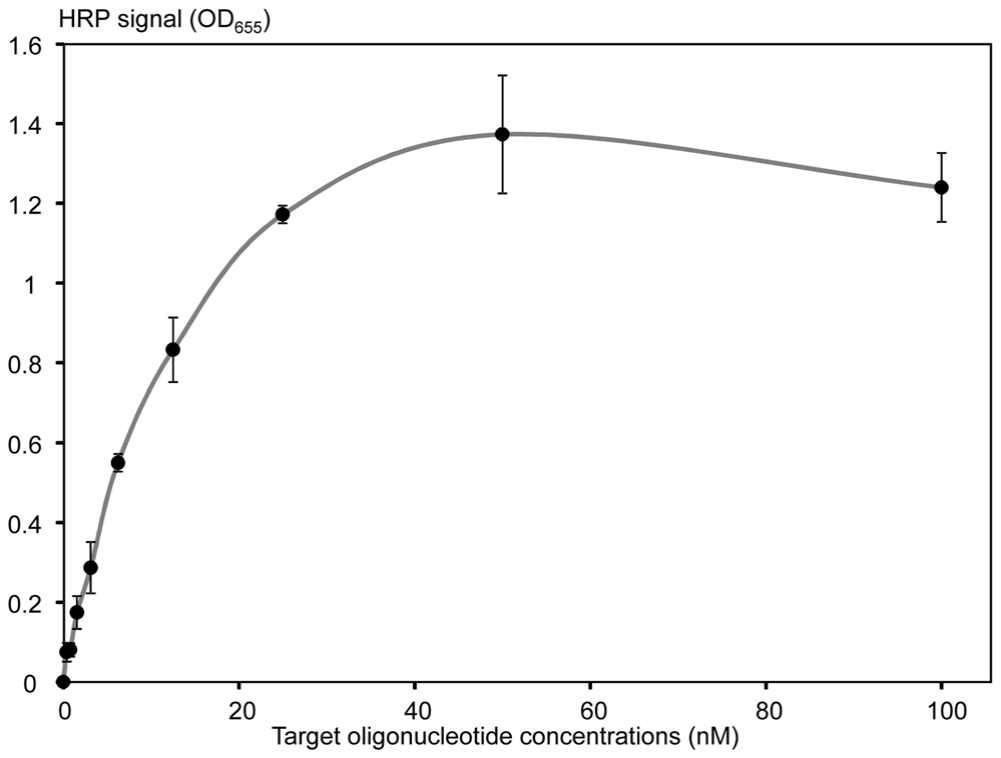
A typical calibration curve of the restriction enzyme assay generated with a 40-mer oligonucleotide target AMC-40-mer (fully complementary to the MCA-BG probe). X-axis shows concentrations (nM) of the target oligonucleotide. Y-axis shows the restriction enzyme generated HRP signal that was quantified by the blue color formation as measured by the OD_655_. The signal values were background-corrected by subtracting the signal generated by the negative control with no target oligonucleotide added. The experiments were performed in triplicate to generate mean values (black circles) and standard deviations (shown with error bars).

This scheme was first tested using a 40-mer oligonucleotide target, fully complementary to the MCA-BG probe and forming a target-probe duplex as shown in Fig. 1E. Serial target dilutions ranging from 0 to 100 nM were used for calibration curve analysis, and showed a typical logarithmic signal dependent upon concentration, with the limit of detection around 1 nM (Fig. 2). Signal saturation was observed at 50 nM concentrations (Fig. 2), likely due to the limited amount of available HRP conjugate immobilized on the ELISA well surface. Use of a high surface capacity carrier, *i.e.* streptavidin-agarose beads, resulted in approximately 10-fold increase of the higher detection limit (data not shown).

### Effect of sequence alterations on the restriction enzyme assay

**Figure 3:**
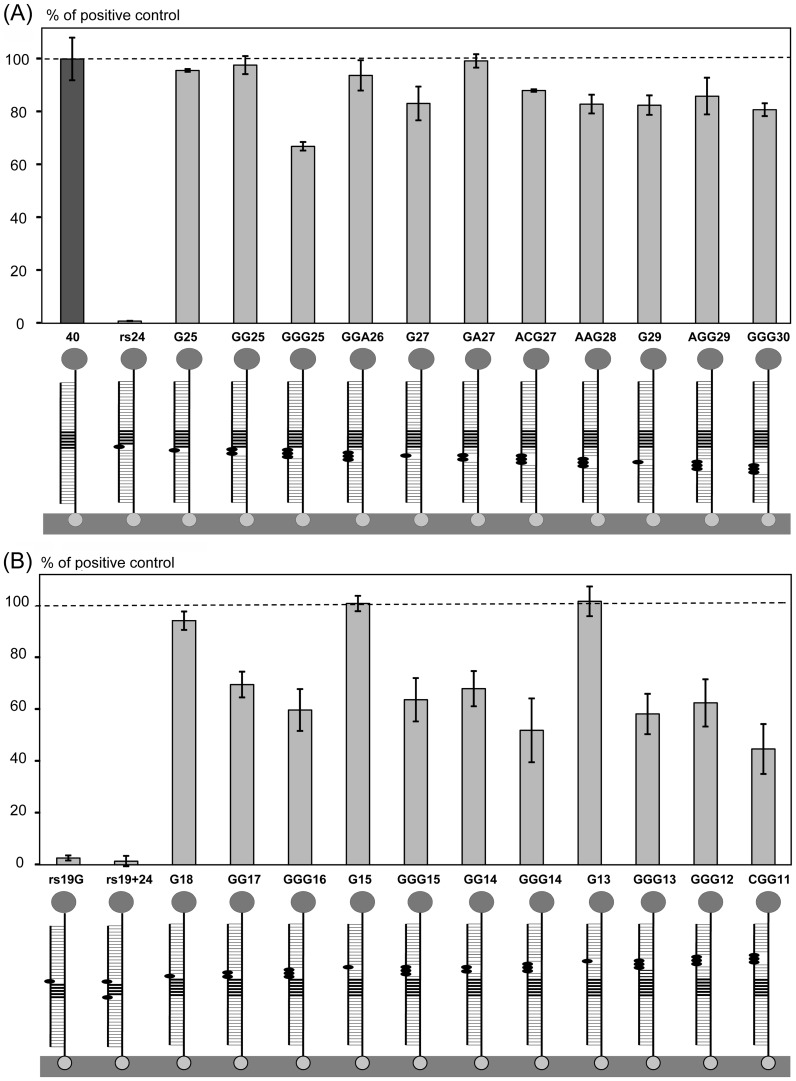
Effect of point mutations introduced into the target sequence. (**A**) Single, double and triple mutations were introduced between the target center and the 3′ end corresponding to the surface-immobilized terminus of the target-probe duplex. (**B**) Mutations were introduced between the target center and the 5′ end corresponding to the end of the target-probe duplex that was free in solution. HRP signals (bars) are expressed as the percentages of the fully cognate positive control (dark grey bar 40, for 40-mer). Target-probe duplexes shown below the bars consist of (1) the probe attached to the streptavidin-modified surface with biotin (bottom) and conjugated to HRP (top), and (2) a 40-mer target with 1–3 mutations shown with black ovals. The *Bgl*II restriction site is indicated with thick horizontal lines. Targets are named with ‘rs’ for mutations introduced within the restriction site, otherwise the target name contains the replacement nucleotide (mostly G) and position within the sequence, starting from the 5′ target end. The rs19+24 contained two mutations at the ends of the restriction site. Target oligonucleotide sequences are shown in Table 1.

A large collection of 47 partially-complementary target oligonucleotides was used to evaluate the effect of mutations and non-cognate additions upon the restriction enzyme assay performance (for sequences see Table 1). This analysis provided information about limitations with respect to probe design, and detection of allelic variation. Point mutations (nucleotide replacements with G or A) were introduced into the *Bgl*II restriction site AGATCT, or in the flanking sequences towards the 3′ (Fig. 3A), or 5′ (Fig. 3B) ends of the 40-mer oligonucleotide target. Each target oligonucleotide was assayed in a series of 4 dilutions, with the values summarized as an integrated signal and expressed as a percentage of the positive control (for the details see Materials and Methods). This approach allowed us to compare characteristics of calibration curves via integrated area values.

Three point mutations in the restriction site (two single and one double) reduced the HRP signal to almost zero (Fig. 3). In contrast, single point mutations introduced into the flanking sequences did not affect the HRP signal, even if they were adjacent to the restriction site (Fig. 3). Double, and especially triple, point mutations resulted in HRP signal reduction to between 50–80% of the positive control. Interestingly, the signal reduction was more pronounced when mutations were introduced into the 5′ end (Fig. 3B) of the target compared to those at the 3′ end (Fig. 3A). This may be due to the fact that the 5′ end was free in solution, while the 3′ end was immobilized on the surface. Proximity to the surface may have stabilized the mutated probe-target duplex, allowing more efficient cleavage (Fig. 1E).

### Target length requirements

**Figure 4:**
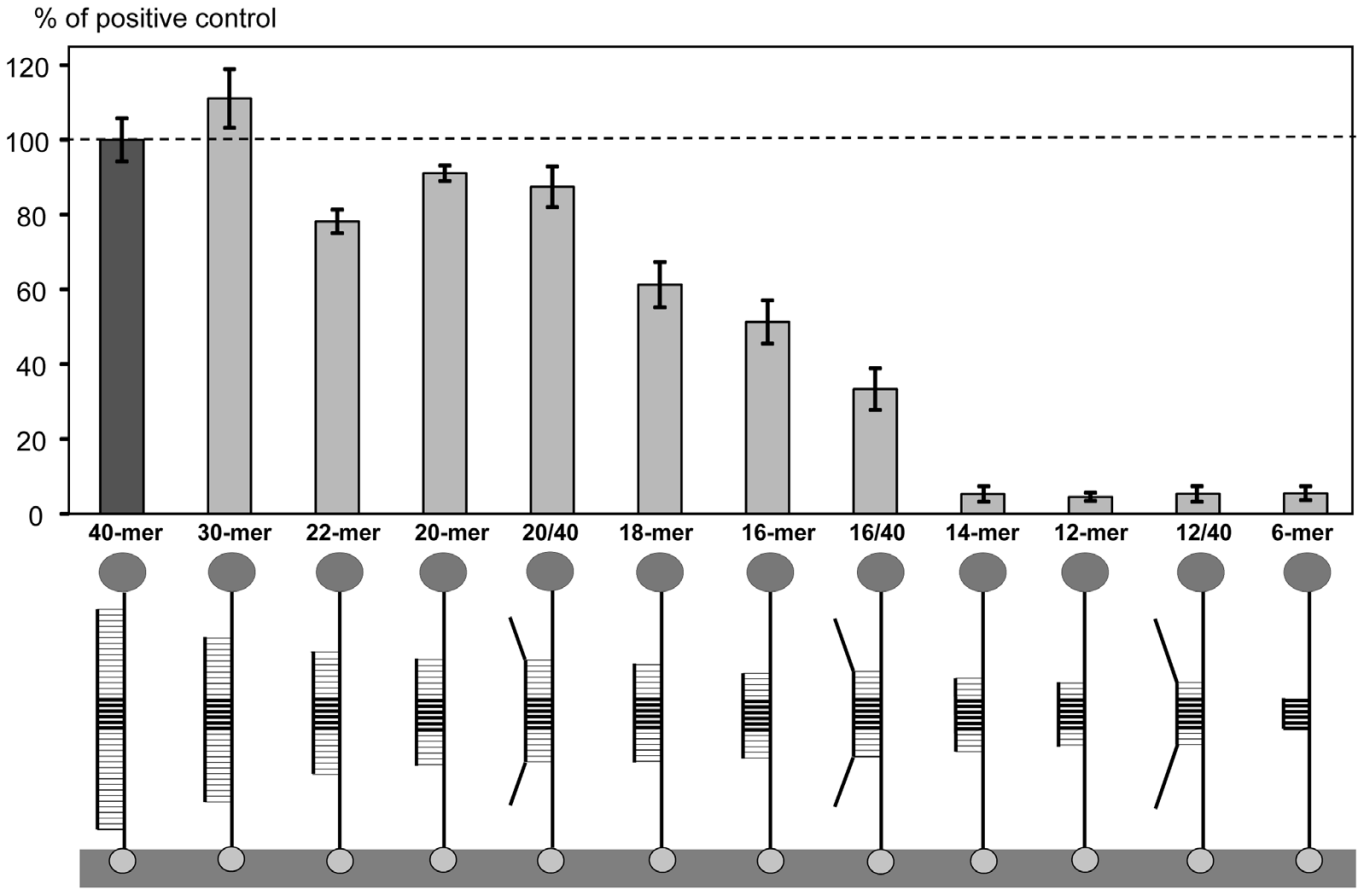
Effect of sequence length on assay. The HRP signals (bars) are expressed as the percentages of the fully cognate positive control (the dark grey bar 40-mer). Target-probe duplexes shown below the bars consist of (1) the probe attached to the streptavidin-modified surface with biotin (bottom) and conjugated to HRP (top), and (2) a target of variable length and end sequence. The *Bgl*II restriction site is indicated with thick horizontal lines. Target-probe duplex designations indicate the complementary sequence length, or fraction of complementary sequence to the total target length. The target oligonucleotide sequences are shown in Table 1.

We designed a series of fully cognate targets of different lengths (from 6 to 30-mer, at 2 nucleotide increments between 12 and 22-mer, Table 1) with the *Bgl*II restriction site positioned at the center. The restriction enzyme assay results were expressed as a percentage of the positive control (40-mer) HRP signal (Fig. 4). The 30-mer target produced a 10% signal increase over the 40-mer (Fig. 4), despite a 3°C lower T_m_ of the 30-bp target-probe hybridization (Table 1). We speculate that this increase was a result of sequence fidelity in the commercial oligonucleotide preparations. In other words, a higher proportion of the full-length sequences were present in the 30-mer compared to the 40-mer preparations.

Further decrease in the target length to 22- and 20-mers resulted in an HRP signal that was approximately 90% of the positive control (Fig. 4). This decrease was probably associated with the 10°C drop in T_m_ for the shorter target-probe duplex (Table 1). However, further reduction in the HRP signal for targets less than 20 nt in length was very dramatic, to approximately 50% of the positive control (Fig. 4). This decrease was much greater than the corresponding decrease in the calculated T_m_ (Table 1). The assay with the 14-mer target yielded an HRP signal of almost zero, which was also the case for the 12- and 6-mer targets (Fig. 4). Thus, a minimum of 16-bp target-probe duplex length was required for significant *Bgl*II cleavage (>50% of the positive control).

Additionally, three partially cognate targets (40-mer) were tested, each containing a fully cognate portion of the sequence immediately surrounding the restriction site (12, 16, or 20-mer) that was flanked with non-cognate ends (Table 1, 12/40, 16/40 and 20/40 targets, respectively). The HRP signals observed for the longest (20/40) and the shortest (12/40) cognate sequences were similar to their fully cognate counterparts (Fig. 4). However, addition of non-cognate ends to the 16-mer apparently destabilized the target-probe hybrids, reducing the signal from 51 to 33% (Fig. 4).

### Effects of restriction site position and non-cognate loop additions

**Figure 5:**
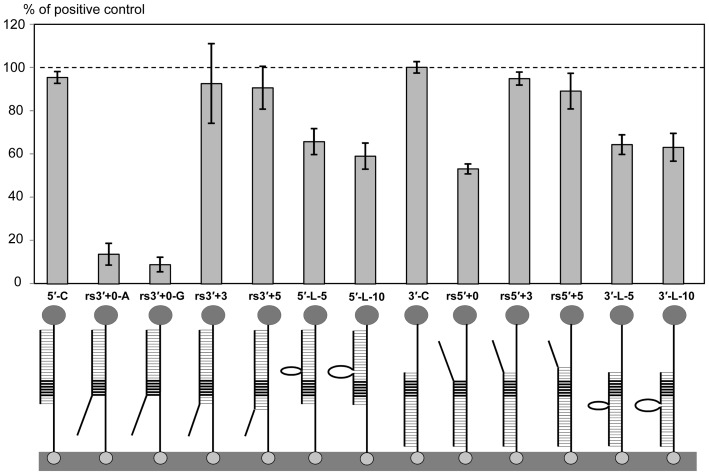
Effects of restriction site positioning within the ds DNA hybrid, and non-complementary loop addition. The HRP signals (bars) are expressed as percentages of the fully cognate positive control (40-mer). Target-probe duplexes shown below the bars consist of (1) the probe attached to the streptavidin-modified surface with biotin (bottom) and conjugated to HRP (top), and (2) a target of variable length, non-complementary ends, and/or loops. The *Bgl*II restriction site is indicated with thick horizontal lines. Target designations are the following: 5′ (or 3′), corresponds to the 5′ (or 3′) ends of the full length positive control; C, control (fully cognate), L, loop (addition of 5 or 10 nucleotides); rs5′ (or rs3′), the end of restriction site to which 0, 3, or 5 (+0, +3, +5) complementary nucleotides were added. For rs3′+0, two targets were prepared that had different non-complementary sequences flanking the 3′-end of the restriction site (rs3′+0-A, rs3′+0-G). The target oligonucleotide sequences are shown in Table 1.

Further analysis was performed to analyze the effects of restriction site positioning within the cognate duplex. Shortening of the target-probe duplex to 25–27 bp and positioning of the restriction site within 3–5 bp from either the 5′- or 3′-end did not significantly decrease the HRP signal (Fig. 5). However, if the restriction site was positioned at 0 nucleotides from the 3′ end of the duplex, the signal decreased dramatically, *i.e.* 9–14% for the two constructs tested (rs3′+0-A and rs3′+0-G, Fig. 5). Interestingly, when the restriction site was positioned at 0 nucleotides from the 5′ end of the duplex, the signal decreased to only 50% (Fig. 5), probably again due to surface immobilization effects on the duplex.

We also tested the effects of inserting relatively long ssDNA loops (5 and 10 nucleotides) into the cognate duplex, which was 25–27 bp length. The loops were positioned 3 nucleotides upstream or downstream from the restriction site. Overall, the effects of loop additions were similar to the triple mutations (Fig. 3), reducing the HRP signal to 60–65% of the positive control (Fig. 5). Thus, as long as the restriction site was at least 3 bp away from the ends of a duplex of >20 bp in length, the assay generated relatively high HRP signals. This was true even if the duplex had additional non-complementary sequences.

### Detection of the *mecA* amplicon

**Figure 6:**
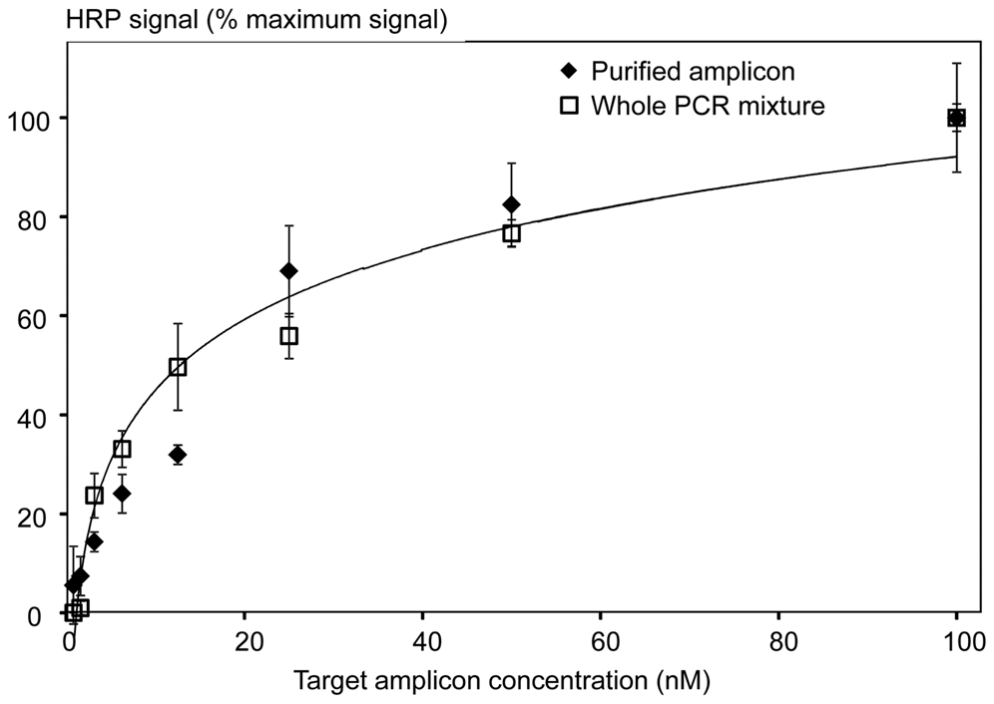
Calibration curves generated with either the purified 196*mecA* amplicon (diamonds) or the unpurified PCR mixture (containing the target amplicon) (squares). The logarithmic trendlines were calculated in Excel, and proved to be identical for the purified and non-purified amplicons.

The restriction enzyme assay was used for detection of the 196 bp *mecA* amplicon [15] as follows. The ds amplicon was generated by PCR using the purified MRSA genomic DNA (strain TCH1516) as a template. The amplicon was heat-denatured to make the target strand available for hybridization to the immobilized probes. Since both target and anti-sense strands were present in the denatured mixture, the strand-to-strand re-association was competing with the target-probe hybridization. Because long incubation times favor hybridization of long over short DNA strands, the assay hybridization time was reduced to 30 min. Overall, the strand re-association resulted in up to 10X lower values of the HRP signal for the ds amplicon compared to the oligonucleotide. Nevertheless, the calibration curve obtained using serial dilutions of the purified 196 bp amplicon (with concentrations ranging from 0 to 100 nM) showed a similar detection limit (approximately 1 nM, Fig. 6) to that observed for the oligonucleotide targets (Fig. 2). A logarithmic dependence of the HRP signal on the target concentration was observed for the full range of amplicon dilutions (Fig. 6). Apparently due to the strand re-association, saturation of probes bound to the plate surface with the amplicon targets was not achieved even at the 100 nM concentration.

Next, the restriction enzyme assay was used for amplicon detection in the unpurified PCR mixture after thermocycling. The same PCR mixture prior to cycling served as the negative control for background subtraction. Use of either purified or non-purified amplicons resulted in nearly identical calibration curves (Fig. 6), with the same logarithmic signal dependence and limit of detection.

**Figure 7:**
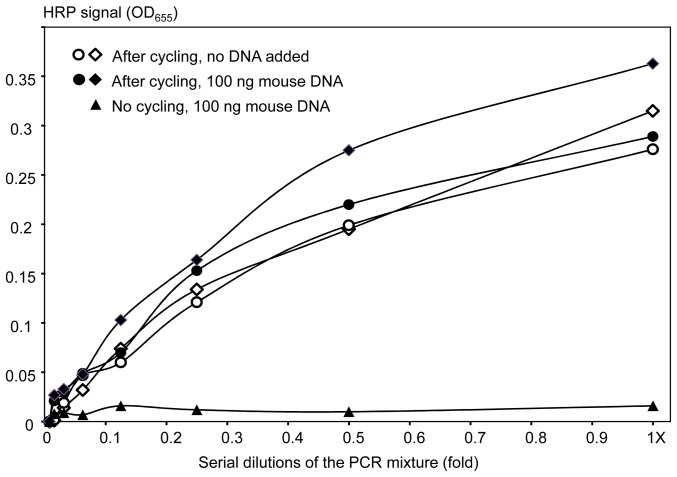
Detection of the non-purified amplicon *mecA* in the presence of a large excess of heterologous (mouse) genomic DNA. Circles and diamonds show replicate experiments performed using the amplicon-containing PCR mixture, closed and open for addition of 100 or 0 ng of mouse DNA, respectively. The triangles show the negative control supplemented with 100 ng of mouse DNA, specifically, dilutions of the whole PCR mixture that were not subjected to thermocycling (no amplicon formation as verified by gel electrophoresis).

To further assess assay specificity, we evaluated the *mecA* amplicon in the presence of a large excess of non-cognate, heterologous DNA. Serial dilutions of the PCR mixture after cycling (containing the *mecA* amplicon) were supplemented with either 0 or 100 ng of mouse genomic DNA (open and closed circles/diamonds in Fig. 7, respectively). Negative control dilutions were prepared using the same PCR mixture prior to cycling (no amplicon) with 100 ng of mouse genomic DNA added, and they produced near zero HRP signal values (Fig. 7, triangles). The results obtained for the PCR mixture containing the amplicon showed almost no difference between the restriction enzyme assays performed in the presence or absence of the mouse DNA. The calibration curves were similar in terms of absolute signal values, the limit of detection and logarithmic nature of the signal dependence on target concentrations (Fig. 7).

Finally, restriction enzyme assays with unpurified PCR mixtures were used for near real-time detection of amplicon formation. We performed qPCR using 0.1 and 1 ng of MRSA genomic DNA as template, and collected aliquots of the PCR mixture every 4 cycles starting from the 8^th^ and ending with the 28^th^ cycle. The aliquots of the initial PCR mixture prior to cycling and at the 35^th^ cycle were used as the negative and positive controls, respectively. The restriction enzyme assay detected the presence of amplicon starting from the 20^th^ and 24^th^ cycle for 1 and 0.1 ng of template, compared to detection by real-time PCR in prior experiments at 16.54±0.17 and 19.92±0.12 cycle, respectively. Thus, the sensitivity of the restriction enzyme assay for amplicon detection was similar to that of the fluorogenic-based qPCR.

## Discussion

### Restriction enzyme assay design

Design of targets for the restriction enzyme assay requires consideration of a number of issues related to probe and restriction enzyme selection. In the case of the *mecA* gene responsible for the antibiotic resistance phenotype of *S. aureus* [15], we started with the following: (1) Recognition sites ≥6 bp with higher complexity (containing all 4 nucleotides) were preferred over shorter sites with lower-complexity (*i.e.* containing only T and A); (2) Sequences flanking the restriction site should have relatively high complexity and an absence of repeats to confer higher specificity; and (3) Stable secondary structure formation should be avoided within probes to promote probe-target hybridization. For the *mecA* amplicon, we selected a 40-mer probe with the *Bgl*II restriction site at the center, which was conjugated to HRP, and the resultant conjugate was attached through biotin to the streptavidin-modified surface of ELISA plate wells. Calibration curve analysis using the fully cognate 40-mer oligonucleotide target showed a clear logarithmic dependence of the signal on the target concentration, with a limit of detection of 1 nM (Fig. 2).

### Minimum length requirements, and effects of sequence alterations and restriction site positioning

Extremely high specificity of cleavage within a recognition sequence has been demonstrated for several class II restriction enzymes. For example, *Eco*RI is shown to bind to the correct recognition site 90000-fold better than miscognate sites that have one incorrect base pair [30]. Consistent with this finding, when the *Bgl*II restriction site was altered by point mutations in our study, restriction was not detected (Fig. 3).

Little is known with respect to requirements by restriction enzymes for the sequences flanking the restriction site. The commercial restriction enzyme manufacturers provide a general guideline of 6 bases on either side of the recognition sequence to ensure efficient cleavage, and mention that different enzymes may have different requirements (http://www.roche-applied-science.com/shop/products/restriction-enzymes-technical-tips). Published data are available only for the commonly studied restrictase *Eco*RI, showing that the three flanking base pairs on either side of the restriction site are essential for cleavage, since their alteration can change the specific enzyme binding constant by as much as 500-fold [25].

We used a collection of 47 mutated target oligonucleotides to characterize the: (i) effects of mutations on hybridization; (ii) requirement for probe-target duplex length; and (iii) requirements for positioning of the recognition site within the target. Single point mutations introduced into the flanking sequences had very limited to no effect on assay outcome. Double and, especially, triple point mutations reduced restriction to 50-80% of the positive control value (Fig. 3), and similar effects were observed for insertions of short single-stranded DNA loops of 5 and 10 nucleotides (Fig. 5). Interestingly, the HRP signal was reduced more for mutations positioned closer to the end of the target-probe duplex that was free in solution, compared to those closer to the surface-immobilized end (Fig. 3). The partially cognate target-probe duplexes may be stabilized by proximity to the surface, thus resulting in partial mitigation of the disruptive effect of mutations. This suggests that our restriction enzyme assay can be used for detection of different allelic variations using a common probe that positions the variable part of the target towards the surface. Conversely, multiple SNP-based allelic variations may be detected using several probes, with the SNP sites positioned close to the solution end of the target-probe duplex to achieve maximum effect on the HRP signal.

Our data indicated that a minimum of 16-bp target-probe dsDNA duplex was required for significant cleavage (30–50% relative to the positive control), with shorter targets producing no signal. This result suggested the assay has very high specificity, because on average in a random DNA sequence, a cognate 16-mer would be observed only once every 4.3 Gbp. Further experiments showed that the exact position of the restriction site within the target-probe duplex did not affect the HRP signal significantly, as long as the site was located 3 or more nucleotides away from the end. Taken together, our experiments defined the restriction enzyme assay requirements for target and probe design. The results indicated that the restriction enzyme assay is highly specific and will be useful for detection of allelic variation in pathogens or other organisms of interest.

### Use of the restriction enzyme assay for detection of PCR products

The optimized assay was also used for detection of PCR amplicons. Since amplicons were double-stranded, a denaturation step was required to separate the target and anti-sense strands. Overall, the maximum HRP signal was about an order of magnitude lower for the amplicon compared to the single-stranded oligonucleotide target. Presumably, this decrease occurred because the competing process of strand re-association reduced the amount of target strand available for hybridization to the probe. Nevertheless, the same limit of detection (1 nM) was observed for both ds amplicons and oligonucleotide targets. Importantly, amplicon purification was unnecessary, as our data showed no difference when we used the purified *mecA* amplicon or the unpurified PCR mixture (Fig. 6). This is consistent with the commercial manufacturer's suggestions that many restriction enzymes may be fully active in a PCR mixture, and therefore suitable for direct use (http://www.roche-applied-science.com/shop/products/restriction-enzymes-technical-tips).

Addition of a large (approximately 10X) excess of heterologous (mouse) genomic DNA to the PCR mixture containing the *mecA* amplicon did not affect the restriction enzyme assay performance (Fig. 7). There may have even been some improvement over results without heterologous DNA additions, especially at low amplicon concentrations, which could be attributed to a decrease in strand re-association by the presence of heterologous DNA [31]. The restriction enzyme assay was also used to follow amplicon formation during PCR thermocycling in near real-time. PCR cycling thresholds were similar between the restriction enzyme assay and the standard fluorescence-based qPCR detection assay. Thus, the restriction enzyme assay may be used in a semi-quantitative format to evaluate PCR product formation, with parallel genotyping and detection of multiple different amplicons from the same unpurified PCR mixture.

## Conclusions

Currently, over 3000 Class II restrictases have been discovered, and approximately 300 are available commercially (https://www.neb.com/products/restriction-endonucleases/restriction-endonucleases). This large collection enables rapid selection of a specific restriction enzyme for practically any relatively long (>100 bp) target of interest. Our experiments provided a defined algorithm for probe design and subsequent testing of a restriction enzyme-probe pair. The amplicon restriction enzyme assay is fast and simple, requiring only target hybridization followed by restriction cleavage, which can be achieved in under 1 hour. The detection costs are lower than those of qPCR, since no fluorescent reagents are used. In the future, detection can be achieved using an electrochemical format simply by changing HRP substrates. The restriction enzyme assays have extremely high selectivity due to the requirement for two biorecognition steps, and thus are able to detect specific sequences in the presence of excess of heterologous DNA. Therefore, the assay can be used for amplicon genotyping in conjunction with various end-point and qPCR applications. Addition of restriction enzyme assays can allow for fast, electrophoresis-free detection and quantification of multiple different genes from the same PCR mixture generated in broad-range PCR. Furthermore, the relative ease of assay design and optimization will facilitate use of the restriction enzyme assay for the genotyping of emerging microbial pathogens.
